# The Antiviral Drug Arbidol Inhibits Zika Virus

**DOI:** 10.1038/s41598-018-27224-4

**Published:** 2018-06-12

**Authors:** Susan L. Fink, Lucia Vojtech, Jessica Wagoner, Natalie S. J. Slivinski, Konner J. Jackson, Ruofan Wang, Sudip Khadka, Priya Luthra, Christopher F. Basler, Stephen J. Polyak

**Affiliations:** 1 Department of Laboratory Medicine, University of Washington, Seattle, Washington USA; 2 Department of Obstetrics and Gynecology, University of Washington, Seattle, Washington USA; 3 Center for Microbial Pathogenesis, Institute for Biomedical Sciences, Georgia State University, Atlanta, USA

## Abstract

There are many emerging and re-emerging globally prevalent viruses for which there are no licensed vaccines or antiviral medicines. Arbidol (ARB, umifenovir), used clinically for decades in several countries as an anti-influenza virus drug, inhibits many other viruses. In the current study, we show that ARB inhibits six different isolates of Zika virus (ZIKV), including African and Asian lineage viruses in multiple cell lines and primary human vaginal and cervical epithelial cells. ARB protects against ZIKV-induced cytopathic effects. Time of addition studies indicate that ARB is most effective at suppressing ZIKV when added to cells prior to infection. Moreover, ARB inhibits pseudoviruses expressing the ZIKV Envelope glycoprotein. Thus, ARB, a broadly acting anti-viral agent with a well-established safety profile, inhibits ZIKV, likely by blocking viral entry.

## Introduction

The recent outbreak of Zika Virus (ZIKV), a flavivirus, in the Americas involved over 47 countries, 20,000 infections, and spread to US Territories (over 500 cases in Puerto Rico and US Virgin Islands) and 148 cases in 38 of the 50 US States (CDC website,^1^).

ZIKV is a member of the *Flaviviridae*, which is related to the mosquito-borne flaviviruses that include Dengue, West Nile, and Japanese encephalitis viruses^2^. A distant relative is the hepatitis C virus, which lies in a novel genus within the *Flaviviridae* called *Hepacivirus*. Upon binding to one or more cell-surface receptors, flaviviruses enter cells via endocytosis. Once the endosomal lumen is acidified, the viral surface glycoproteins undergo a conformational change, which induces fusion of the endosomal membrane with the viral envelope, releasing the viral genome into the cytoplasm. The ZIKV genome is single stranded, positive sense RNA and over 10 kb in length. Like all flaviviruses, the RNA genome encodes a genome-length polyprotein precursor, with proteins arranged in the following order: 5′-C-prM-E-NS1-NS2A-NS2B-NS3-NS4A-NS4B-NS5-3′. The polyprotein is cleaved by cellular and viral proteases to yield the viral structural and non-structural proteins (NS). Structural proteins include Capsid (C), precursor Membrane (prM), Envelope (E), and Non-Structural proteins (NS). Many NS proteins have roles in viral replication and include NS1 (modulates host immunity), NS2A, NS2B, NS3 (protease), NS4A, NS4B, NS5 (polymerase)^3^.

ZIKV is typically transmitted to humans following a bite by an infected mosquito, mainly by *Aedes aegypti*. ZIKV is also transmitted from mother to fetus/newborn and sexually. ZIKV RNA can be found in blood, urine, semen, saliva, amniotic fluid, breast milk and cerebrospinal fluid^4^. Symptoms of ZIKV illness are usually mild, can last for several days to a week, and include fever, rash, joint pain, and conjunctivitis (red eyes) (https://www.cdc.gov/zika/). Despite mild symptoms in most human infections, ZIKV can cause neurological damage in humans and in developing fetuses. For example, ZIKV has been associated with Guillain–Barré syndrome, an autoimmune disease that affects the peripheral nervous system^5^. ZIKV infection of pregnant women is increasingly associated with multiple congenital central nervous system malformations, including microcephaly^6^ and miscarriages^7^. The neurological effects of ZIKV infection are being borne out in animal models^8,9^. New concerns have arisen with the detection of infectious virus in reproductive tissues and sexual transmission of the virus^10^.

Arbidol (ARB, also known as Umifenovir, PubChem CID 131410), is a synthetic antiviral drug developed 30 years ago to combat seasonal influenza virus^11^. Since that time, ARB has been shown to inhibit viruses from many different families including orthomyxo^12^, paramyxo^13^, picorna^14^, bunya^15^, rhabdo^16^, reo^13^, toga^17^, hepadna^18^, hepaci^11,19–22^, and filoviridae^23^. In the current study, we demonstrate the antiviral potential of ARB against Zika virus.

## Materials and Methods

### Starting Material

**Figure 1:**
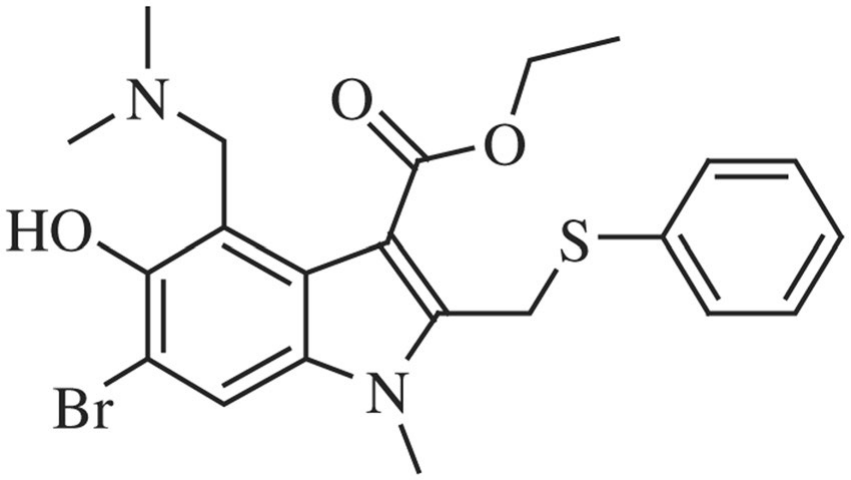
ARB Structure. Compound ID in PubChem: 131410.

ARB (Fig. 1) was synthesized commercially. Purity and structure was confirmed as described^23^. ARB was dissolved in ethanol or DMSO.

### Cells and Viruses

A549 (human lung epithelial)^24^, Huh7.5.1 (human hepatoma)^25^ and Vero (African green monkey kidney)^26^ cells were grown as described^23,27,28^. HEK-293T cells (human embryonic kidney) were grown in high-glucose Dulbecco’s modified Eagle’s medium (DMEM) supplemented with 10% Serum Plus II (Sigma), 1% Hepes, 100 U/ml penicillin, and 100 µg/ml streptomycin. Ugandan (MR766), Puerto Rican (PRVABC59), Panamanian (PA 259249), and Mexican (MEX 2–81) Zika virus isolates were obtained from Robert B. Tesh (University of Texas Medical Branch). A Brazilian isolate (KX811222.1) from Fortaleza was obtained from Justin Roby and Michael Gale Jr. (University of Washington). A ZIKV isolate from Honduras was obtained from ATCC (ZKV-1848). Viral stocks were generated by infecting Vero cells at a multiplicity of infection (MOI) of 0.01 and resulting supernatants were titered on Vero cells by standard plaque assay^29^. Briefly, 2 × 10^5^ Vero cells were plated the day before in 12 well plates. Virus stocks were diluted 1:10 serially in media. After removing media from cells, 300 μl of diluted viral stock per well was added to triplicate wells per dilution. The inoculum was left on cells for 1 hr at 37 °C, removed, and cells were covered in 1 ml of 1X Overlay Medium (consisting of equal parts of 2% Methocell MC (Sigma) in water and 2X media (2X MEM, 10% FBS, 100 U/ml penicillin, 100 µg/ml streptomycin, 1 mM sodium pyruvate). Five to six days later, plates were inspected by inverted microscopy for evidence of cytopathic effect (CPE). Overlay medium was then removed and the monolayer washed two times with PBS. The monolayer was fixed with 1 ml 4% paraformaldehyde in PBS at room temperature for 20 minutes. Cells were stained with 0.5 ml of 2% crystal violet solution for about 5 minutes, before removing stain and washing with PBS. Plates were then washed by immersing in a plastic container filled with tap water. Plaques were counted to derive the titer in PFU/ml by the formula: Average number of plaques/(volume of inoculum plated × dilution factor).

### Epithelial cell cultures

Cultures of primary, untransformed epithelial cells were generated from *ex-vivo* tissues, discarded following routine vaginal repair surgeries or hysterectomies, and cultured as described^30–32^. Cells were maintained in F medium (3:1 [v/v] F12 [Ham]-DMEM [Life Technologies], 5% fetal calf serum [Gemini Bio- Products], 0.4 μg/ml hydrocortisone [H-4001; Sigma], 5 μg/ml insulin [700-112 P; Gemini Bio-Products], 8.4 ng/ml cholera toxin [227036; EMD Millipore], 10 ng/ml epidermal growth factor [PHG0311; Life Technologies], 24 μg/ml adenine [A-2786; Sigma], 100 U/ml penicillin, and 100 μg/ml streptomycin [Life Technologies]). Cells were routinely cultured in the presence of irradiated (6000 Rad) 3T3-J2 feeder fibroblasts and 10 μM of Rho kinase inhibitor Y27632 (1254; Enzo Life Sciences). Feeder cells were detached first by 1 min treatment with 10 ml Versene (Life Technologies), followed by 5 min treatment with trypsin/EDTA (Life Technologies) to dislodge epithelial cells. 100,000 epithelial cells were plated in each well in 1 ml of medium in 12 well plates 1 day prior to infection.

### ARB Treatment and Infections

Arbidol was usually added to cells before virus infection, except for time of addition studies where the drug was added before, during, and after virus infection. Please refer to figure legends for the details of when ARB was added. Zika virus was added to cells at MOIs of 0.01 to 3 and infectivity was measured by various assays between 24–72 hours post-infection.

### Measuring Zika Virus Infection

For infection of Huh7.5.1 cells, Zika virus protein expression was measured by Western blot analysis using the LI-COR Odyssey CLx imaging system. Rabbit antibodies to ZIKV E (GTX133325), NS5 (GTX133312), Capsid (GTX133317), or NS1 (GTX133304) proteins, or mouse anti-ZIKV-NS1 (GTX634159) were obtained from GeneTex. Goat-anti-Actin (sc-1616), mouse anti-vinculin (sc-73614), or mouse anti-cofilin E8 (sc-376476) antiserum (all from Santa Cruz Biotechnology) were also used to detect cellular proteins and confirm equal protein loading across all samples. Secondary antibodies to the goat, mouse, or rabbit primary antibodies were labeled with infrared dyes, permitting detection with the Odyssey CLx. Dyes designated as 800 produce green emission, while dyes designated 680 produce red emission. The secondary antibodies used were donkey anti-goat DyLight680 or donkey anti-goat DyLight800 (SA5-10090, SA5-10092, Fisher), donkey anti-rabbit DyLight680 (SA-510042, Fisher), goat anti-rabbit DyLight800 (SA-510036, Fisher), goat anti-mouse IRDye680RD (925-68070, LI-COR), and goat anti-mouse IRDye800CW (925-32210, LI-COR). For some blots, combinations of antibodies were used to facilitate multiplex detection of viral and cellular proteins. Blots were stripped using NewBlot Nitro Stripping Buffer (928-40030, LI-COR) following manufacturer’s instructions. For all Western blots, Image Studio (LI-COR) software was used to obtain Western blot images, using default instrument settings of resolution at 169 μm and scan quality set to lowest. Any image manipulations were manually applied equally across the entire image and were applied equally to controls. The manipulations consisted of adjusting the brightness and contrast and/or flipping the image to obtain the proper orientation. The original Western blot images are included in the Supplementary Information. Using Image Studio software, rectangles of identical size and area were drawn around viral and cellular protein bands to obtain pixel intensities, which were exported into excel. All protein bands were normalized to cellular protein levels. ARB-treated samples were then normalized to the ethanol solvent control. Final pixel intensities were expressed as percent of ethanol control by first dividing the actin-normalized ARB treated sample pixel intensity by the actin-normalized ethanol solvent control pixel intensity. This fraction was then multiplied by 100.

For infection of A549 cells, ZIKV protein expression was measured by immunostaining and quantification of an HRP-conjugated secondary antibody using TMB substrate by measuring absorbance at 650 nm^28^. Cells were pretreated with ARB with four-fold serial dilution (40–0.001 μM) for one hour, followed by infection with ZIKV MR766 at an MOI of 1 in the presence of drug. Two-hours later, viral inocula were removed and fresh medium containing drug was added back to cell cultures. Forty-eight hours post-infection, the cells were fixed with ice-cold methanol, and washed with assay buffer (PBS with 2% nonfat milk and 0.1% Triton X-100). The cells were then incubated with anti-flavivirus group antigen E antibody (1:4000 dilution, clone D1-4G2-4-15, Sigma) for two hours at room temperature. Cells were washed three times with assay buffer and incubated with anti-mouse horse radish peroxidase (HRP) conjugated secondary antibody (1:4000 dilution, in assay buffer) for one hour at room temperature. The cells were further washed three times with assay buffer and then incubated with TMB (100 μl) (Rockland Immunochemicals, PA) for 30 min at room temperature. TMB is the substrate for HRP, which converts TMB to a blue color. The intensity of the color is proportional to the amount of viral antigen and was measured by spectrometry at 650 nm using EnVision (Perkin Elmer).

For ZIKV infection of primary vaginal and cervical epithelial cells, Western blots were used to measure ZIKV proteins as described above. Progeny virus production was also measured by plaque forming unit (PFU) assays after diluting supernatants at least 1:100. ZIKV RNA genome copies were also quantified by digital droplet RT-PCR with a Biorad QX200 system. RNA from cell cultures was extracted using the Qiagen RNeasy kit according to manufacturer’s instructions. An equivalent amount of RNA for all conditions was reverse transcribed using random hexamers, diluted 1:5, and used as template for digital droplet PCR using Biorad ddPCR supermix for probes, according to manufacturer’s instructions. Copies of ZIKV RNA were normalized to the cellular housekeeping gene RPP30. PCR primers and probe for ZIKV were ZKV F (5p-CCGCTGCCCAACACAAG), ZKV R (5p-CCACTAACGTTCTTTTGCAGACAT), ZKV probe (5p-AGCCTACCTTGACAAGCAGTCAGACACTCAA).

### Zika pseudovirus particles

ZIKV pseudovirus was made by harvesting culture supernatant of HEK-293T cells co-transfected with a DNA-launched WNV replicon expressing EGFP^33^ (kindly provided by Theodore Pierson, National Institute of Allergies and Infectious Diseases, MD, USA) and a plasmid expressing ZIKV C-PrM-E (kindly provided by Oscar Burrone and Jose Luis Slon Campos, International Centre for Genetic Engineering and Biotechnology, Trieste, Italy) as described^34^. Vero cells were pretreated with ARB for 2 h prior to pseudovirus infection, harvested 24 h after infection, fixed with paraformaldehyde and analyzed by flow cytometry on a BD LSR Fortessa. EGFP data were derived from gating on live cells, based on forward/side scatter characteristics. The percentage of dead cells was comparable between all samples and generally less than 10%.

### Cytoxicity Testing

Cytotoxicity of ARB on Huh7.5.1, Vero, and primary human vaginal or cervical epithelial cells was evaluated by measuring cellular ATP levels with a commercial kit (ATPlite assay, Perkin Elmer). Cytotoxicity of ARB on A549 cells was measured using the CellTiter-Glo reagent (Promega).

## Results

**Figure 2:**
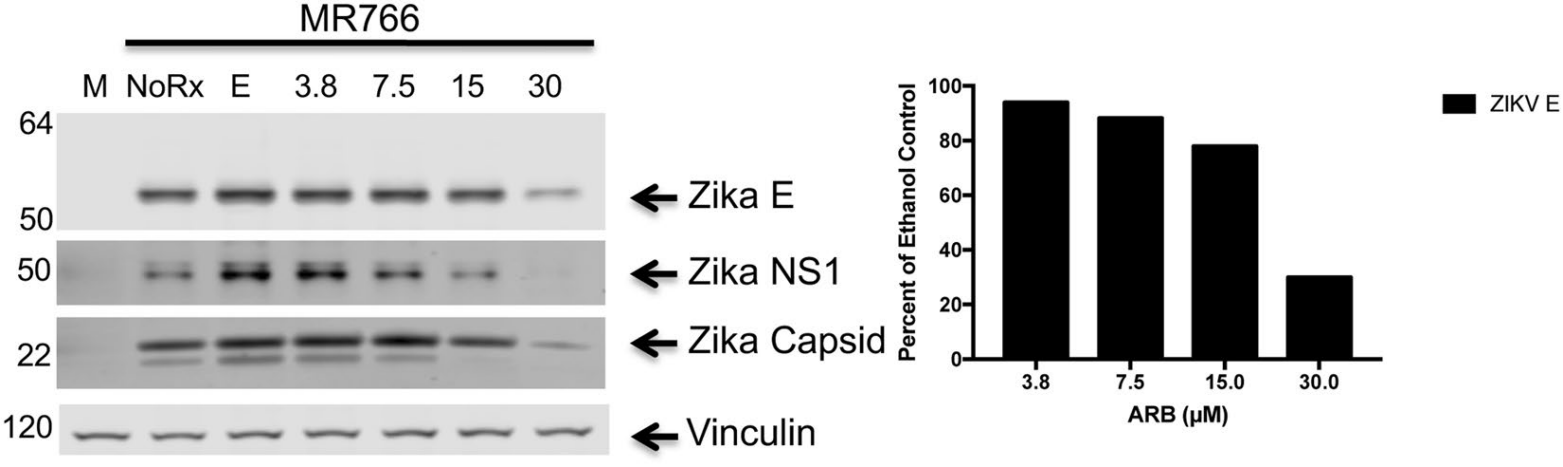
ARB causes dose-dependent inhibition of ZIKV protein synthesis. Vero cells were treated with ethanol solvent control (E) or 3.8, 7.5, 15, or 30 μM of ARB for 30 minutes prior to infection with ZIKV MR766 at an MOI of 0.01. The images on the left side of the figure depict ZIKV Envelope (E), NonStructural 1 (NS1), and Capsid protein expression, detected by Western blot analysis of protein lysates harvested 72 hours post-infection. The image is a composite of the following antibody probings: the blot was first probed with rabbit anti-ZIKV-E (detected with anti-rabbit 800 labeled secondary antibodies) and mouse anti-vinculin antiserum (detected with anti-mouse 680 labeled secondary antibodies). The blot was stripped and reprobed with rabbit anti-ZIKV-Capsid (detected with anti-rabbit 800 labeled secondary antibodies) and mouse anti-ZIKV-NS1 antiserum (detected with anti-mouse 680 labeled secondary antibodies). M = mock infected cells. No Rx = ZIKV-infected cells without further treatment. E = ZIKV-infected cells treated with ethanol solvent control. Molecular weight markers are as indicated. Right bar graph shows the relative pixel intensity across the 4 doses of ARB used in this assay. Original blot images are shown in Supplemental Information.

Figure 1 depicts the structure of ARB, an indole-based compound. Figure 2 demonstrates that ARB inhibits the infection of Vero cells by the Ugandan (MR766) isolate of ZIKV, as indicated by decreased viral protein synthesis. Asian lineage virus isolates circulating in the world including Puerto Rican (PRVABC59), Brazilian (KX811222.1), Cambodian (FSS13025), Panamanian (PA259249), and Mexican (MEX2-81) isolates were also inhibited by ARB (Supplemental Figure S1).

**Figure 3:**
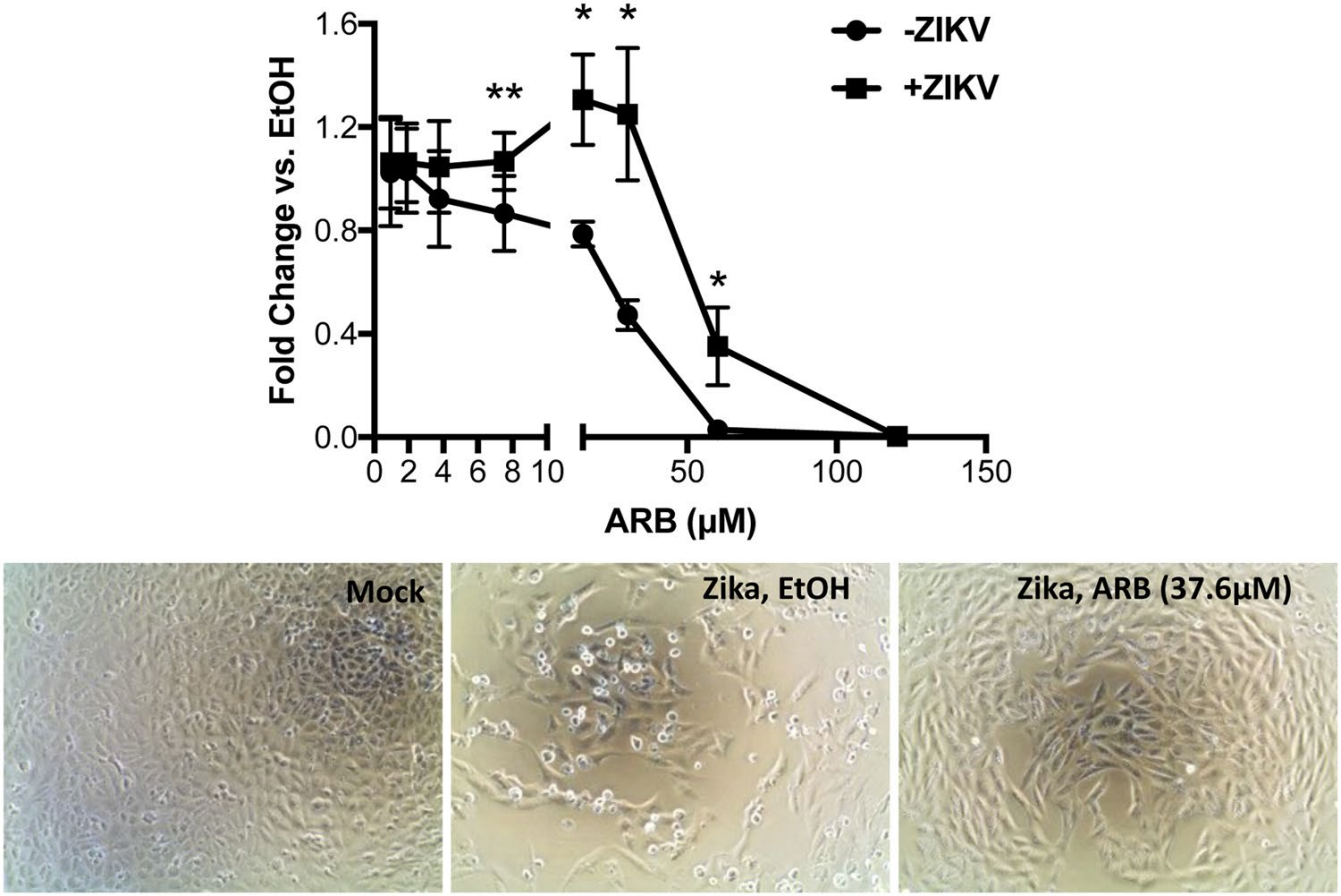
Arbidol protects cells from ZIKV-induced cell death. Vero cells were treated with ethanol solvent control or 0.9, 1.8, 3.8, 7.5,15, 30, 60, or 120.3 µM of ARB immediately prior to infection with ZIKV (Brazil, Fortaleza) at an MOI of 0.01. Control cultures were not infected. Cellular ATP levels were measured 72 hours post-infection by ATPlite assay (Perkin Elmer). (**A**) Fold change in ATP levels in ARB-treated versus ethanol solvent control. *p = 0.02; **p = 0.07, from Student’s T tests. Data are means ± standard deviations of three replicates. (**B**) Light microscopy images of mock and ZIKV-infected cultures, treated with ethanol or ARB.

We also measured the cytotoxicity profile of ARB on Vero cells in the presence and absence of ZIKV infection. Zika infection is cytolytic to Vero cells and killed many cells after a 3-day infection. When the magnitude of cellular ATP reduction in ARB-treated cells was compared to cells treated with ethanol (the solvent for ARB), ARB treatment prevented the reduction in ATP levels (i.e. cell death) during Zika virus infection in Vero cells (Fig. 3A). Microscopically, Zika virus-infected cultures had clear evidence of reduced cell numbers and cytopathic effect (CPE; Fig. 3B, middle panel) as compared to non-infected (i.e. mock) cultures (Fig. 3B, left panel). ARB treatment protected against virus-induced CPE (Fig. 3B, right panel). The data suggest that ARB partially protects cells from ZIKV-induced cell death. These results were not unique to Vero cells as ARB-treated human hepatoma Huh7.5.1 cells were also protected from ZIKV-induced CPE and exhibited reduced ZIKV protein production (Supplemental Figure S2).

**Figure 4:**
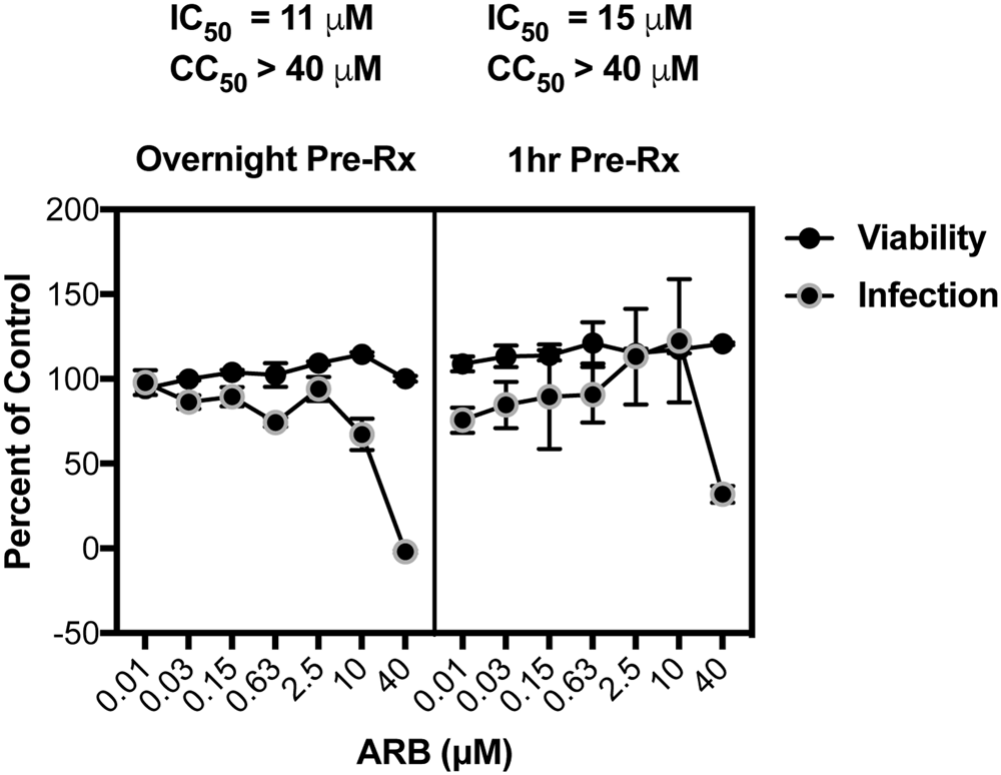
ARB inhibits ZIKV in A549 cells. Cells were pretreated with various doses of ARB either 1 hour before ZIKV infection (RIGHT) or overnight (LEFT) prior to infection with ZIKV MR766 (MOI = 1). Virus was added to cells in the presence of drug, and two-hours later, viral inocula were removed. Medium containing fresh ARB at the respective doses was then added back to cells. Infection was monitored 48 h post infection by immune-staining cells with pan flavivirus anti-E protein antibody 4G2. The expression of protein antigen was quantified using anti-mouse HRP-TMB method by measuring absorbance at 650 nm. Viability was measured by CellTiter-Glo reagent (Promega). The solvent control (i.e. ethanol) was set at 100% for the infection and viability assessments and is reflected on the Y-axis. Data are means ± standard deviation of three replicates.

**Figure 5:**
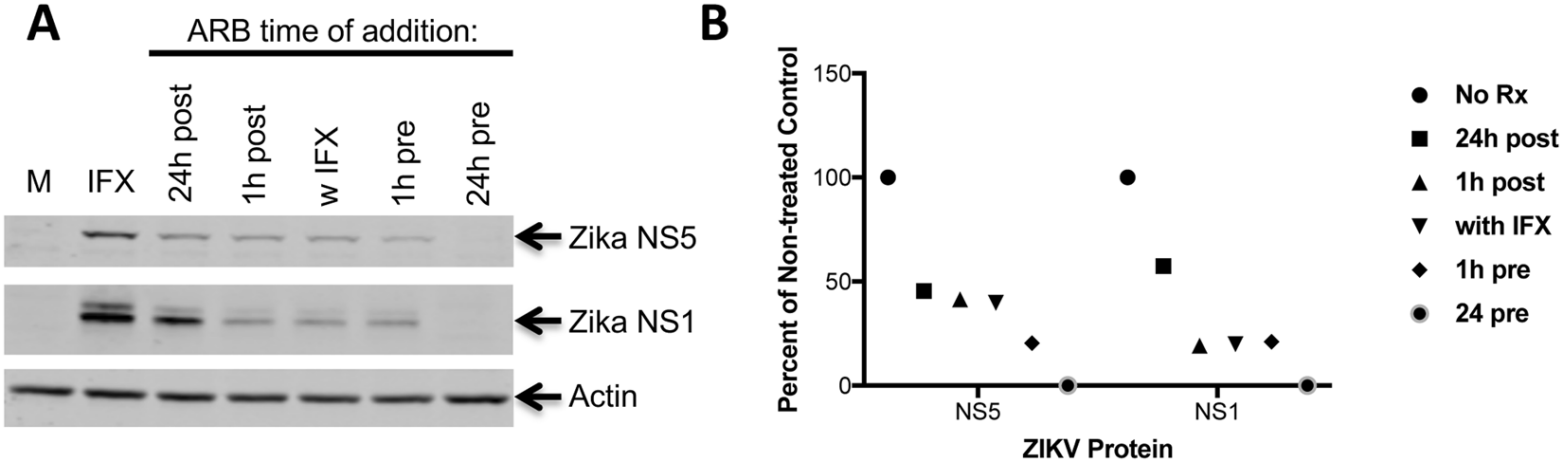
ARB likely inhibits an early step in the ZIKV lifecycle. Human hepatoma Huh7.5.1 cells were infected with ZIKV MR766 at an MOI of 0.02. ARB (15μM) was added before (“pre”), at the same time (“w IFX”), or after infection (“post”) at the indicated times. (**A**) ZIKV NS5 and NS1 proteins were detected by Western blot analysis after 48 hours of infection. The image is a composite of the following antibody probings: the blot was first probed with rabbit anti-ZIKV-NS5 (detected with anti-rabbit 800 labeled secondary antibodies) and goat anti-actin antiserum (detected with anti-goat 680 labeled secondary antibodies). The blot was then stripped and reprobed with rabbit anti-NS1 (detected with anti-rabbit 680 labeled secondary antibodies). “M” denotes mock-infected cells, while “IFX” denotes infected cells not treated with ARB. (**B**) Normalized pixel intensity of NS5 and NS1 protein expression with the different ARB treatments. Original blot images are shown in Supplemental Information.

We further tested ARB against ZIKV infection of human A549 cells and asked whether overnight pre-treatment with ARB provides better antiviral effects against ZIKV as compared to when ARB is added 1 hour before infection. Figure 4 demonstrates that ARB inhibits ZIKV at a concentration that inhibits ZIKV infection by 50 percent, IC_50_, of 11 μM and a concentration that kills 50 percent of cells, CC_50_, of >40 μM when cells are pretreated overnight. In comparison ARB’s IC_50_ is 15 μM when cells are pretreated for 1 hr, with a CC_50_ of >40 μM The data suggest that overnight pre-treatment of cells provides somewhat better activity against ZIKV. Since ARB is known to block entry of HCV and EBOV^16,23^, we performed time of addition experiments (Fig. 5). Addition of ARB to cells 24 hours before ZIKV infection caused the greatest suppression of infection, while adding ARB 1 hour before, at the same time as, and 1 hour after infection also robustly suppressed infection. Addition of ARB to cells at 24 hours post-infection was not as potent as the other times of ARB addition, but still suppressed virus infection by about 50%. The data indicate that ARB is most effective at suppressing ZIKV infection when cells are pretreated with the drug, suggesting that ARB inhibits an early step in the ZIKV lifecycle. In addition, ARB might also impact later steps in the virus life cycle.

**Figure 6:**
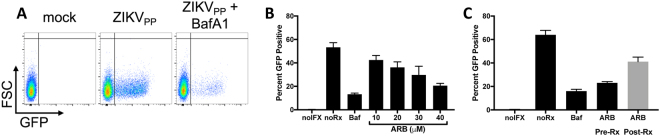
Arbidol inhibits ZIKV_PP_ infection. (**A**) ZIKV pseudoviral particles (ZIKV_PP_) were produced by co-transfecting cells with ZIKV C-PrM-E and a West Nile virus replicon expressing EGFP. Vero cells were infected with ZIKV_PP_ in the presence of Bafilomycin A1 as indicated. EGFP was measured by flow cytometry. (**B**) Effect of ARB on ZIKV_PP_ infection of Vero cells. Cells were pretreated with ARB for 2 hours prior to infection with ZIKV_PP_, and ARB was also present during virus adsorption, and when the inoculum was removed and fresh media added to cells. (**C**) Comparison of 40 µM ARB on ZIKV_PP_ infection when the drug is present throughout the experiment (i.e. during the 2 hour cell pretreatment, adsorption, and post-adsorption; labeled as “pre-Rx”) versus when ARB is added to cells only after the virus inoculum is removed; labeled as “post-Rx”). EGFP was measured 24 hours post-infection. no-IFX = mock-infected cells; no-Rx = infected cells treated with EtOH solvent; Baf = bafilomycin A. Data are means ± standard deviation of three replicates.

To focus on virus entry mediated by the ZIKV Envelope glycoprotein, we generated pseudovirus particles with Zika virus Envelope and Capsid packaging a West Nile virus replicon encoding an EGFP reporter^34^. Infection of Vero cells with the pseduovirus particles yielded EGFP positive cells, and entry was blocked by Bafilomycin A1, a known inhibitor of endosomal acidification (Fig. 6A). The infectivity of these particles on Vero cells was prevented in a dose-dependent manner by pretreatment of cells with ARB (Fig. 6B). ARB was most effective when the drug was present throughout the infection process, including during pre-treatment of cells, during virus inoculation, and when the inoculum was removed and replaced with fresh medium (Fig. 6C, ARB treated sample labeled as “pre-Rx”). In contrast, ARB was less effective at suppressing pseudovirus infection, when the drug was added to cells after the virus inoculum was removed (Fig. 6C, ARB treated sample labeled as “post-Rx”). These data reinforce the concept that ARB blocks an early step in the ZIKV life cycle, and may also have post-entry effects.

**Figure 7:**
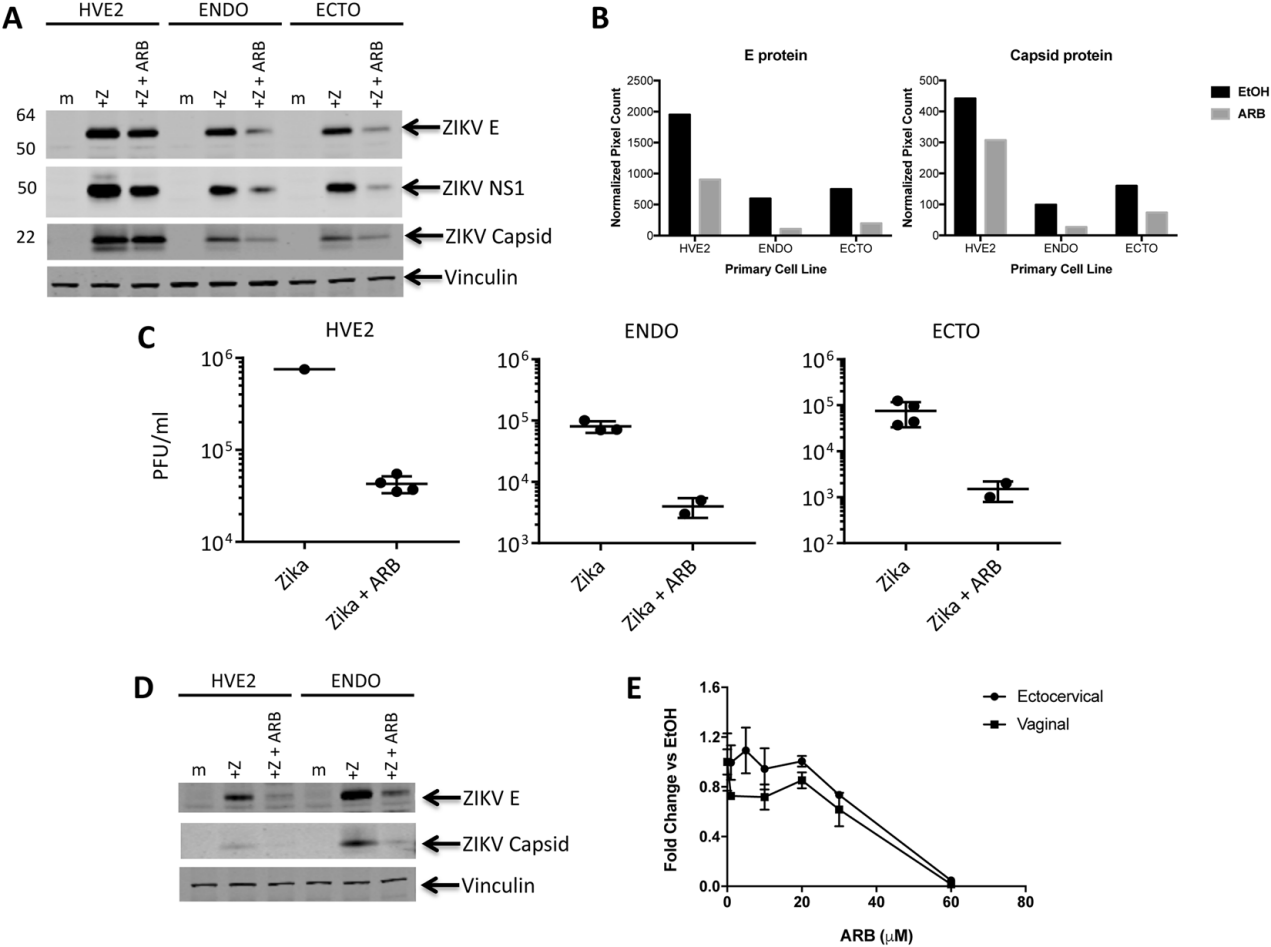
Arbidol inhibits Zika virus infection of primary vaginal and cervical epithelial cells. (**A**) Western blot of human vaginal epithelial cells (HVE2), endocervical (ENDO), or ectocervical (ECTO) cells that were mock (m) infected or infected with ZIKV MR766 (+Z) at a MOI of 1 and treated without or with 20 μM ARB for 72 hours. Expression of ZIKV E, NS1, and Capsid proteins are denoted, as well as expression of the cellular protein Vinculin. The image is a composite of the following antibody probings: a blot was first probed with rabbit anti-ZIKV-NS1 (detected with anti-rabbit 800 labeled secondary antibodies) and mouse anti-vinculin antiserum (detected with anti-mouse 680 labeled secondary antibodies). A separate blot of the same protein samples was probed with rabbit anti-ZIKV-E (detected with anti-rabbit 680 labeled secondary antibodies). The same blot was then stripped and reprobed with mouse anti-vinculin antiserum (detected with anti-mouse 680 labeled secondary antibodies) and rabbit anti-ZIKV-Capsid (detected with anti-rabbit 800 labeled secondary antibodies). (**B**) Quantitation of ZIKV protein intensity in ethanol or ARB-treated cells. (**C**) Infectious virus production by cells treated with ethanol versus ARB. Culture supernatants were harvested at 72 hours post infection, diluted 1:10,000, and titered on Vero cells. Results are expressed as plaque forming units per ml (PFU/ml). (**D**) ARB inhibits infection of primary human vaginal and cervical epithelial cells by ZIKV strain 1848, an Asian lineage isolate. Cells were infected and treated as in panel A. The image is a composite of the following antibody probings: a blot was first probed with rabbit anti-ZIKV-E (detected with anti-rabbit 800 labeled secondary antibodies) and mouse anti-vinculin antiserum (detected with anti-mouse 680 labeled secondary antibodies). The blot was then stripped and reprobed with rabbit anti-ZIKV-Capsid antiserum (detected with anti-rabbit 800 labeled secondary antibodies). Expression of ZIKV E and Capsid proteins are denoted, as well as expression of the cellular protein Vinculin. (**E**) Cytotoxicity profile of ARB in primary human vaginal and ectocervical cells. Cells were treated with ethanol (EtOH) or 1, 5, 10, 20, 30, 60 μM ARB for 72 hours before viability was measured using ATPlite assay. Numbers represent fold change relative to ethanol treated triplicate cultures. Error bars represent standard deviation. Original blot images are shown in Supplemental Information.

ARB efficacy in primary cell lines was evaluated by infecting primary human vaginal and cervical epithelial cells with ZIKV MR766 in the presence and absence of ARB pretreatment. Figure 7A shows that primary vaginal epithelial cells (HVE2) and primary endocervical (ENDO) and ectocervical (ECTO) cells were robustly infected by ZIKV. ARB treatment (20 μM) resulted in significant suppression of ZIKV protein synthesis (Fig. 7A,B). The yield of infectious virus from ARB-treated cells that were infected by ZIKV was reduced by 10, 10, and 100 fold in HVE2, ENDO, and ECTO cells, respectively (Fig. 7C). ARB also inhibited infection of cells by ZIKV strain 1848, an Asian lineage isolate (Fig. 7D). Critically, the dose of ARB used in these studies (20 μM) was not toxic to either cell type (Fig. 7E). Finally, ZIKV RNA expression in primary vaginal and cervical epithelial cells from different donors infected by MR766 and 1848 virus isolates was also suppressed by ARB (Supplemental Figure S3).

## Discussion

We show herein that ARB caused dose-dependent inhibition of multiple ZIKV isolates of both the African and Asian lineages in multiple cell lines, including primary human vaginal and cervical epithelial cells. ARB protected against ZIKV-induced CPE, and multiple lines of data suggest that ARB potentially blocks multiple steps in the ZIKV lifecycle, with a major effect on virus entry into cells.

How does ARB inhibit so many enveloped viruses? ARB is an indole-based molecule (Fig. 1) able to form supramolecular arrangements through aromatic stacking interactions with selective amino-acid residues of proteins (phenylalanine, tyrosine, tryptophan)^22^. As such, ARB may impair several steps in the life cycle of viruses including virus attachment to cells, fusion of viral and cellular membranes during virus entry^17,21,22,35–37^, clathrin-mediated endocytosis^16^, or virus replication on intracellular membranes, such as membranous webs^20,38^. However, the bulk of the data coalesce on a model whereby ARB targets viral glycoproteins to prevent the fusion of viral membranes with endosomal membranes during virus entry: (1) the crystal structure of ARB bound to the Influenza virus hemagglutinin (HA) fusion protein shows that ARB prevents HA membrane fusion by blocking low pH-induced conformational changes in the protein^39^; (2) ARB inhibits HCV glycoprotein-mediated fusion of viral membranes with model endosomal membranes^11,19–22^; (3) ARB inhibits entry of EBOV and ZIKV pseudoparticles mediated by EBOV-GP^23^ and ZIKV-E viral fusion proteins (this report); (4) ARB resistant viruses selected *in vitro* have mutations in viral fusion proteins^17,40^; (5) several drugs were recently found to bind to a pocket in the native EBOV GP^41^. Clearly, additional work is required on this interesting compound, including structure-guided optimization of ARB against specific virus families^42^.

Clinical success of ARB for ZIKV infection will require sufficient drug levels in target cells and tissues, without toxicity. The recommended oral dose of ARB for treatment of influenza in humans is 200 mg three times daily^43^, but single doses of up to 800 mg have been administered without adverse effects^44^. The maximal plasma concentration (C_max_) and area under the concentration–time curve (AUC) after the standard single ARB dose in humans is approximately 0.9–1.5 µM and 4–6 µM/hr^43–45^, which is in the range of *in vitro* antiviral activity of ARB against many viruses including influenza virus, EBOV, ZIKV, and HCV. Granted, some viruses show inhibition by ARB *in vitro* at doses very close to the plasma exposure (EBOV, IC_50_ = 2.7 µM^23^), while some viruses showing inhibition at higher *in vitro* doses (ZIKV in A549 cells, IC_50_ = 11 µM; this report). It is possible that the dose of ARB for inhibiting a particular virus depends primarily on how tightly the compound engages viral glycoproteins^42^. An 800 mg dose elicits a C_max_ and AUC of 4 µM and 24 µM/hr^44^, which is well within the range of ARB inhibition of most viruses *in vitro*. PK studies in humans indicate plasma C_max_ of ARB is reached within 60–90 minutes, with a half-life (t_1/2_) of 17–21 hours in Russian subjects^11^, with possible faster clearance in Chinese subjects (t_1/2_ = 6–7 hours) in some^45,46^, but not all studies^44^. In mouse studies, doses up to 600 mg/kg/day have been studied without toxicity^13,47–49^, and doses of 90 and 180 mg/kg/day show significant suppression of Influenza H1N1 viral loads, pathology, and mortality^50^. Moreover, allometric scaling^51^ provides non-linear extrapolation of the recommended daily human ARB dose (600 mg) to an oral mouse dose of 120 mg/kg, which is well within the tolerability and efficacious doses of ARB. ARB appears to be safe and without teratogenic effects in pregnant women^52,53^. Thus, in many human and animal studies, ARB has a reasonable pharmacokinetic profile and is well tolerated (reviewed in^11^).

Sexual transmission of ZIKV is well established^10,54^. It is also plausible to speculate that sexually transmitted virus may increase the risk of infection of the placenta and fetus in pregnant women, as compared to mosquito borne transmission. Therefore, infection and replication in the female genital tract are important to consider during assessment of any potential anti-ZIKV therapeutics. We show here that ARB strongly impairs ZIKV in primary cells of the female genital tract and reduces the production of progeny virions. This suggests that ARB would have high activity in cells likely to be targeted during sexual transmission of ZIKV and may be able to prevent local viral replication and systemic spread.

ARB is one example of a drug that should be considered for drug repurposing for virus outbreaks against ZIKV and Ebola virus. Other studies aimed at repurposing of existing drugs have found that bortezomib and mycophenolic acid (MPA) strongly inhibit ZIKV^55^. However, these drugs are immunosuppressive and potentially teratogenic^55^, so their utility in treating ZIKV infection in pregnant women is untenable. Since ARB appears to be safe for pregnant women^52,53^, repurposing the drug with other anti-ZIKV drugs may provide urgently needed options for clinical management of ZIKV disease and its consequences on human health.

## Electronic supplementary material

Supplementary Materials and Figures
