# Supplementary material for: The Antiviral Drug Arbidol Inhibits Zika Virus
Source: Sci Rep. 2018 Jun 12;8:8989. doi: 10.1038/s41598-018-27224-4 (PMC5997637; doi:10.1038/s41598-018-27224-4)
Supplement: Supplementary file 1 — Supplementary Materials and Figures [file 41598_2018_27224_MOESM1_ESM.pdf]

The Antiviral Drug Arbidol Inhibits Zika Virus

Susan L. Fink[1], Lucia Vojtech[2], Jessica Wagoner[1], Natalie S.J. Slivinski[1], Konner J. Jackson[1], Ruofan Wang[2], Sudip Khadka[3], Priya Luthra[3], Christopher F. Basler[3], Stephen J. Polyak[1]

[1] Department of Laboratory Medicine, University of Washington; [2] Department of Obstetrics and Gynecology, University of Washington, [3] Center for Microbial Pathogenesis, Institute for Biomedical Science, Georgia State University.

SUPPLEMENTAL MATERIAL

Original Western Blot Images for Figures 2, 5, and 7

Figure 2 Original Blot Images

First probing: Vinculin and ZIKV E

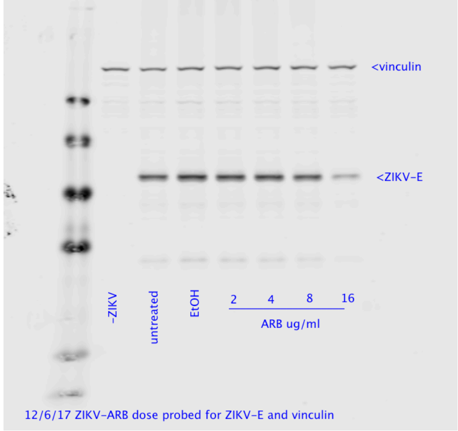

Second probing: ZIKV NS1 and Capsid

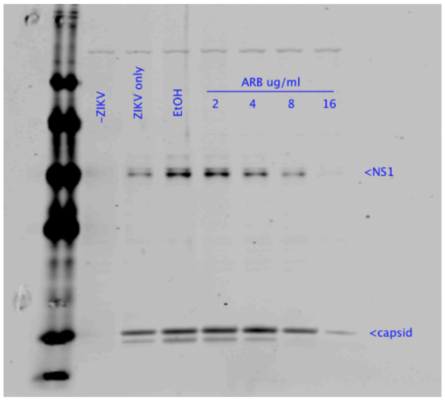

Figure 5 Original Blot Images

First probing: ZIKV NS5 & Actin

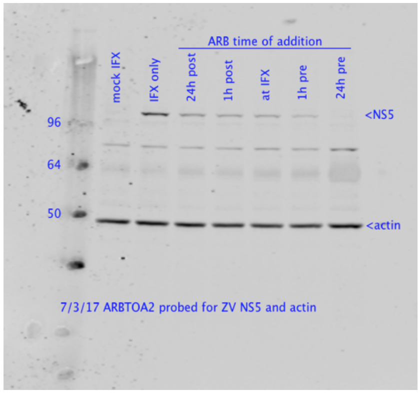

Second probing: ZIKV NS1

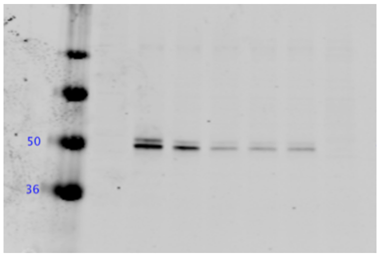

Figure 7A Original Blot Images

Fig 7A: Blot 1 probing: Vinculin and ZIKV NS1

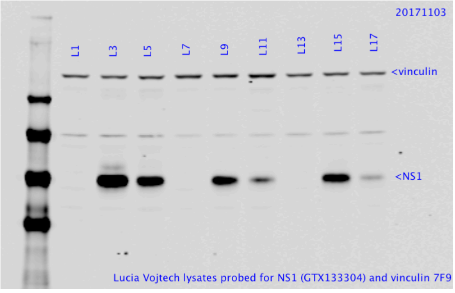

Fig 7A: Blot 2 first probing: ZIKV E

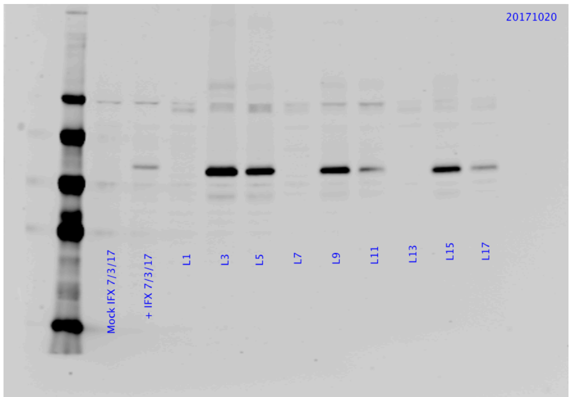

Fig 7A: Blot 2 second probing: Vinculin and ZIKV Capsid

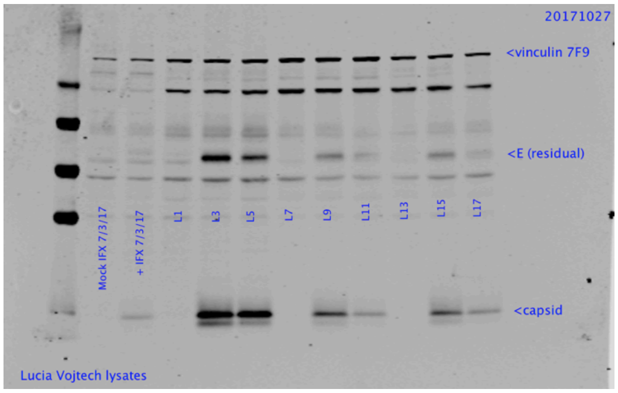

Figure 7D Original Blot Images

First probing: ZIKV E and Vinculin

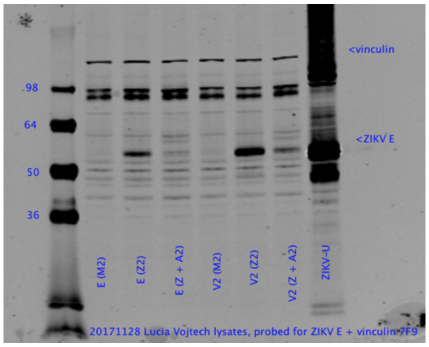

Second probing: ZIKV Capsid

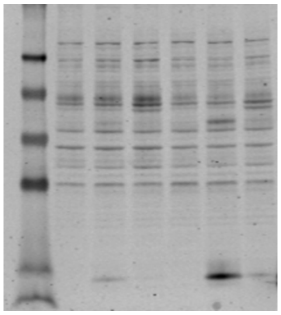

Capsid

Supplemental Figures

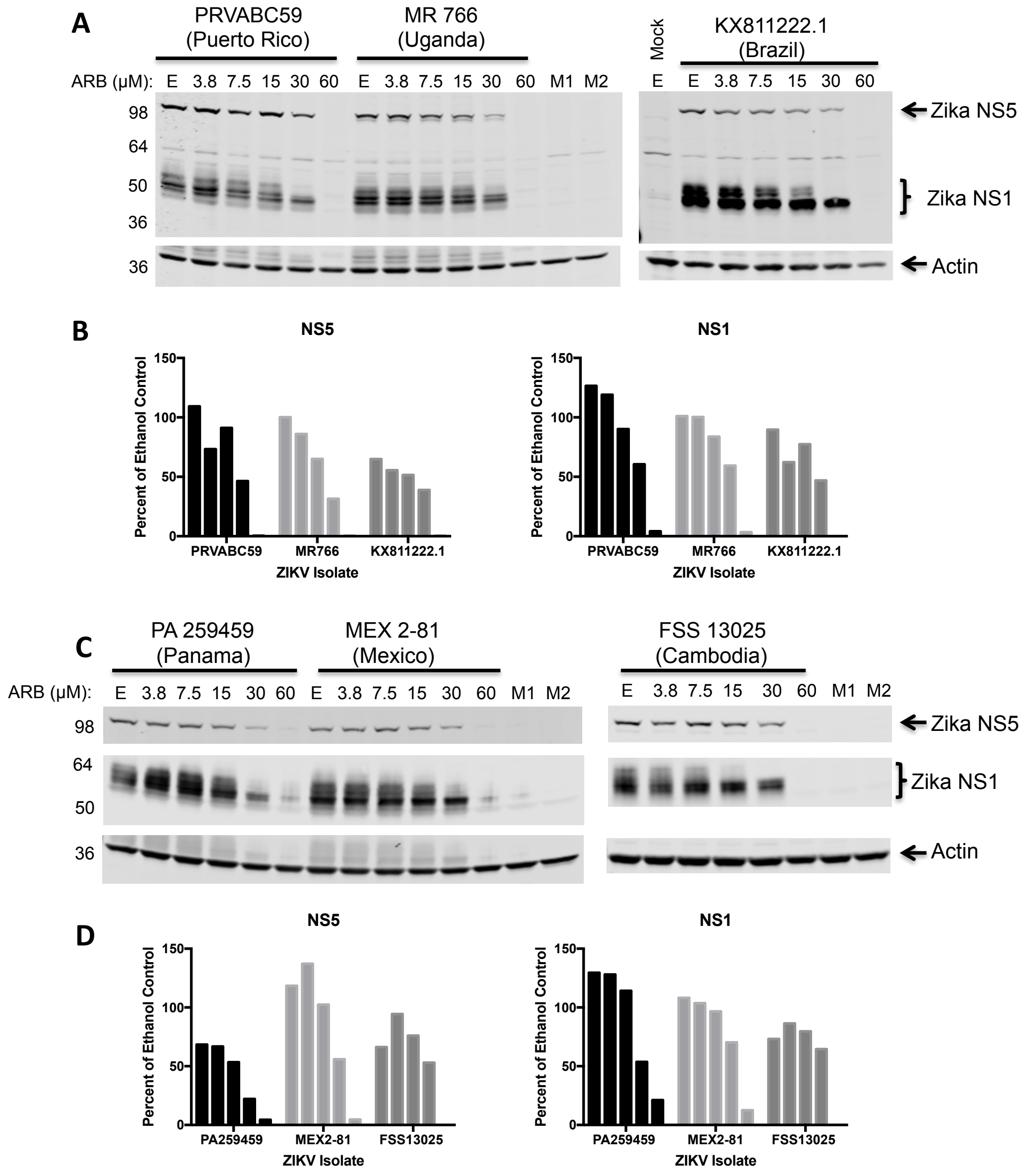

Figure S1 – ARB inhibits an African and multiple Asian ZIKV isolates. Vero cells were treated with ethanol solvent control (E) or 3.8, 7.5, 15, 30, or 60 μM of ARB for 30 minutes prior to infection with the indicated isolates of ZIKV at an MOI of 0.01. M1 = mock infected cells treated with ethanol solvent control. M2 = mock infected cells treated

with 60 μM ARB. Molecular weight markers are as indicated. Panel A, ARB suppression of Puerto Rico, Uganda, and Brazil virus isolates. Panel C, ARB suppression of Panama, Mexico, and Cambodia virus isolates. Panels B and D, bar graphs below each blot show relative pixel intensities across the 5 doses of ARB for each virus isolate. The images represent composites of the following antibody probings: PRVABC59 and MR766 samples were run on the same gel and the blot was first probed with rabbit anti-ZIKV-NS1 (detected with anti-rabbit 800 labeled secondary antibodies), stripped and probed a second time with rabbit anti-ZIKV-NS5 antiserum (detected with anti-rabbit 680 labeled secondary antibodies), and stripped and probed a third time with goat anti-actin (detected with anti-goat 800 labeled secondary antibodies). PA259459 and MEX2-81 samples were run on the same gel the blot was first probed with rabbit anti-ZIKV-NS1 (detected with anti-rabbit 800 labeled secondary antibodies) and goat anti-actin (detected with anti-goat 680 labeled secondary antibodies). The blot was stripped and probed with rabbit anti-ZIKV-NS5 antiserum (detected with anti-rabbit 680 labeled secondary antibodies). FSS13025 samples were first probed with rabbit anti-ZIKV-NS5 (detected with anti-rabbit 800 labeled secondary antibodies) and goat anti-actin (detected with anti-goat 680 labeled secondary antibodies). The blot was stripped and probed with rabbit anti-ZIKV-NS1 antiserum (detected with anti-rabbit 680 labeled secondary antibodies). KX811222.1 samples were probed with a triplex of rabbit anti-NS5 and NS5 antiserum (detected with anti-rabbit 680 labeled secondary antibodies) plus goat anti-actin (detected with anti-goat 800 labeled secondary antibodies). Original blot images are found below.

Original Blot Images for Figure S1

PRVABC59 (left), MR766 (right)

First probing: ZIKV NS1

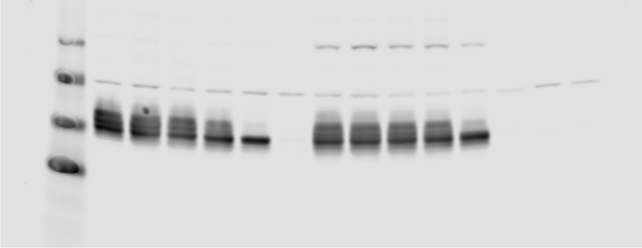

NS1

Second probing: ZIKV NS5

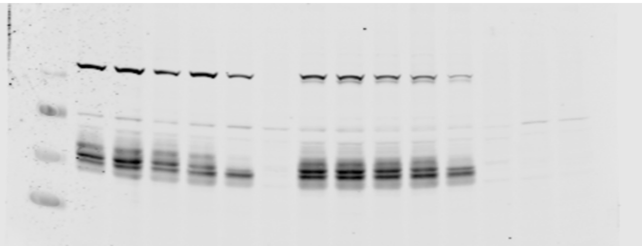

NS5

Third probing: actin

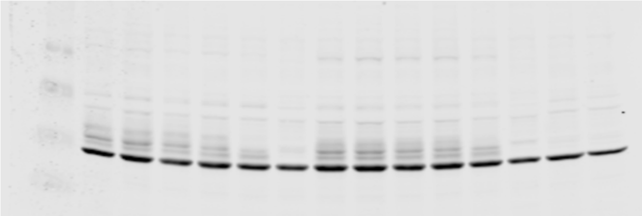

Actin

PA259459 (left), MEX2-81 (right)

First probing: ZIKV NS1 and actin

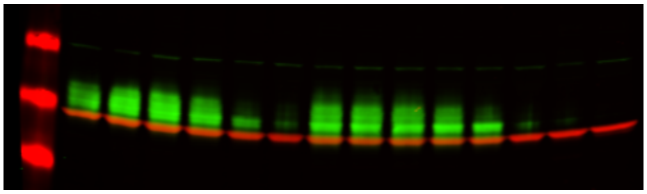

NS1
Actin

Second probing: ZIKV NS5

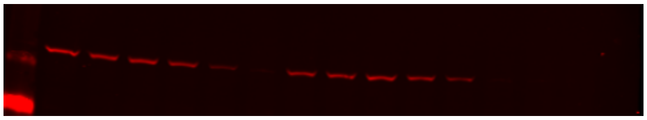

Original Blot Images for Figure S1

FSS13025

First probing: ZIKV NS5, actin

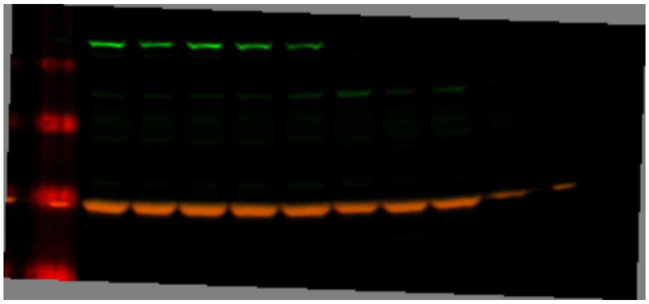

Second probing: ZIKV Capsid

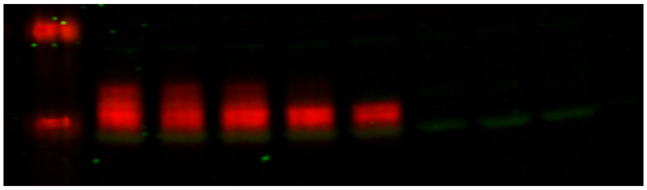

KX811222.1

First probing: ZIKV NS5, ZIKV NS1, and actin

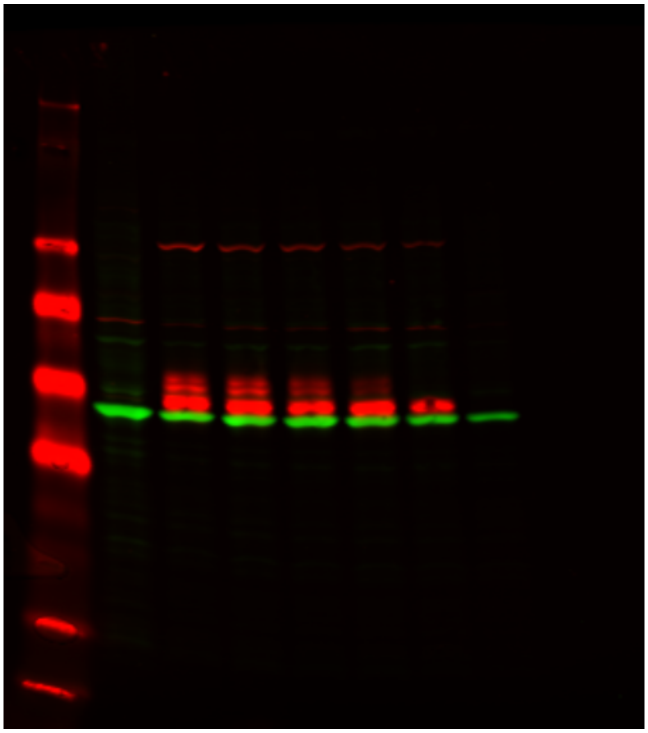

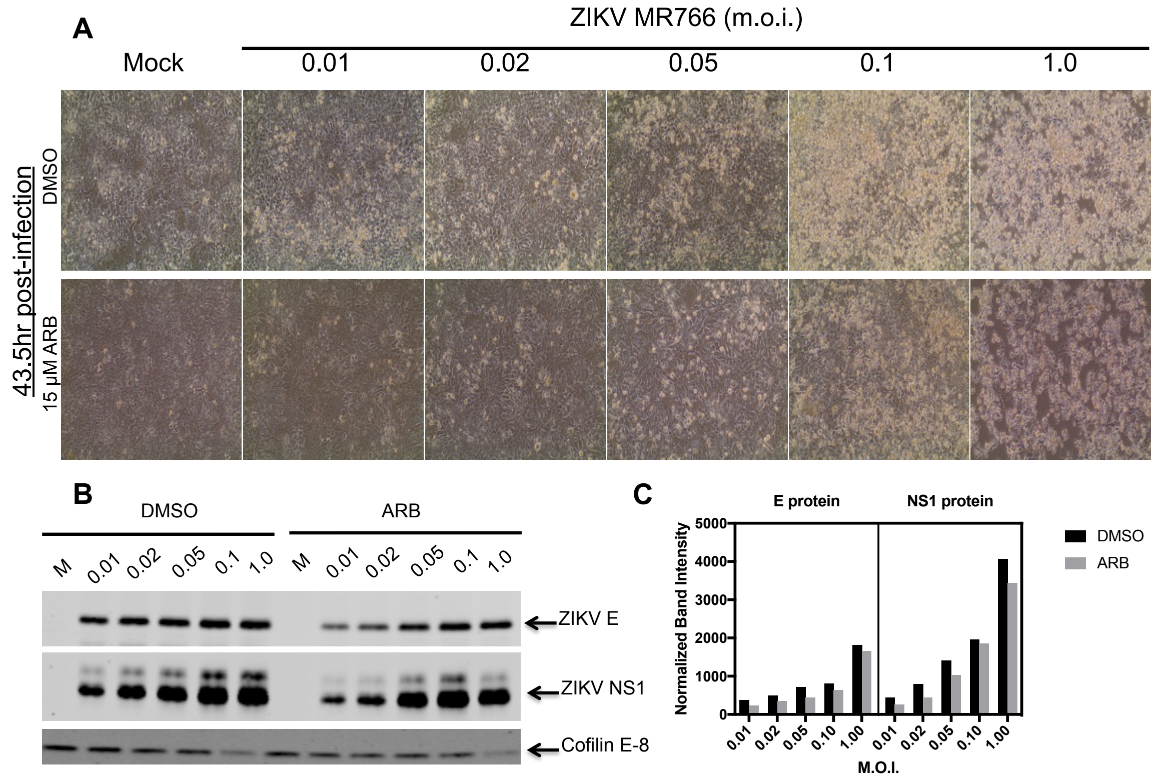

Figure S2 – ARB inhibits ZIKV replication and virus-induced CPE in human hepatoma Huh7.5.1 cells. Huh7.5.1 cells were infected at the indicated MOIs with ZIKV MR766 for 1.5 hours. Viral inocula were removed, cells were washed with PBS, and fresh media containing DMSO solvent control or 15 µM ARB was added to cells. A, light microscopy of mock and ZIKV-infected cultures. B, western blot of ZIKV E and NS1 and cellular protein Cofilin E-8 expression. The image is a composite of the following antibody probings: the blot was first probed with rabbit anti-ZIKV-E (detected with anti-rabbit 800 labeled secondary antibodies). The blot was stripped and reprobed with mouse anti-Cofilin E-8 (detected with anti-mouse 680 labeled secondary antibodies) and rabbit anti-ZIKV-NS1 antiserum (detected with anti-rabbit 800 labeled secondary antibodies). C, Normalized band intensity of western blot data. Original blot images are shown below.

Original Blot Images for Figure S2

First probing: ZIKV E

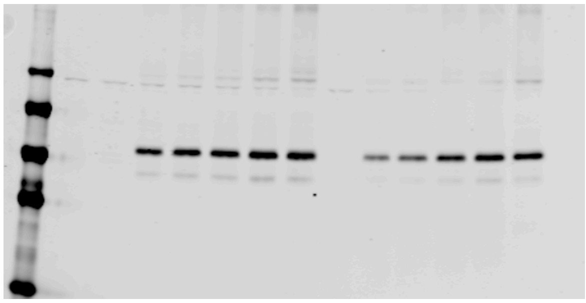

Second probing: ZIKV NS1 and cofilin E-8

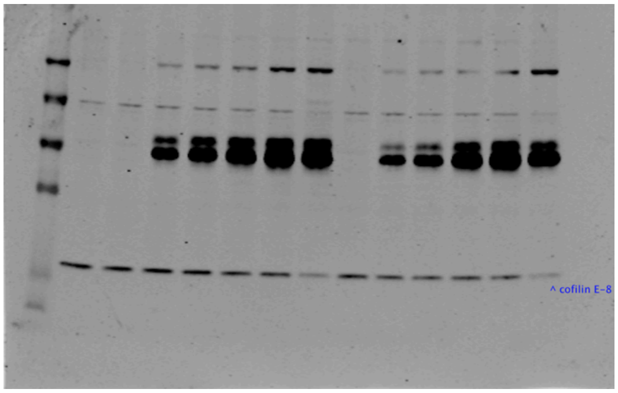

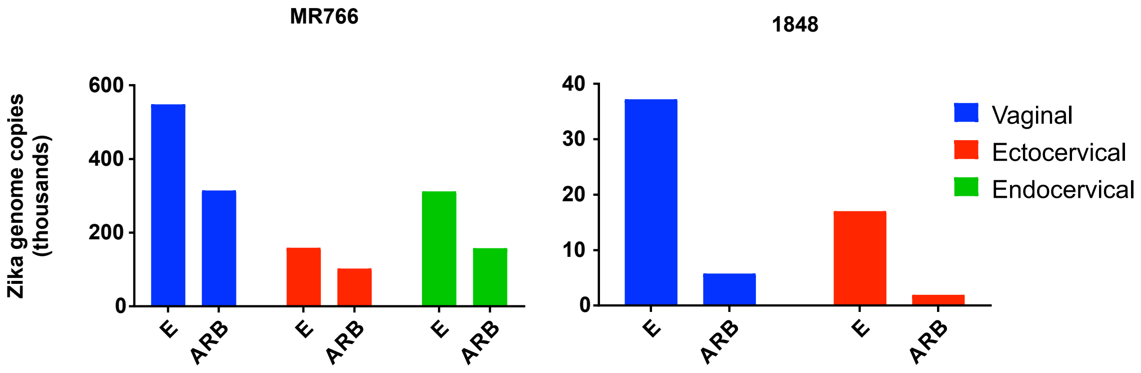

Figure S3: ARB inhibits ZIKV RNA accumulation in primary human vaginal and cervical epithelial cells. Cells were cultured as described in the Material and Methods and infected with ZIKV strains MR766, an African lineage isolate, or 1848, an Asian lineage isolate, at a MOI of 1 for 72 hours, in the presence and absence of 20 μM ARB. RNA was extracted, reverse-transcribed and ZIKV genomes measured by droplet digital PCR.
